# Low-Intensity MR-Guided Focused Ultrasound Mediated Disruption of the Blood-Brain Barrier for Intracranial Metastatic Diseases

**DOI:** 10.3389/fonc.2018.00338

**Published:** 2018-08-28

**Authors:** Ying Meng, Suganth Suppiah, Shanan Surendrakumar, Luca Bigioni, Nir Lipsman

**Affiliations:** ^1^Division of Neurosurgery, Sunnybrook Health Sciences Centre, Toronto, ON, Canada; ^2^Hurvitz Brain Sciences Research Program, Sunnybrook Research Institute, Toronto, ON, Canada

**Keywords:** focused ultrasound (MRgFUS), blood brain barrier (BBB) disruption, neuro-oncology–surgical, intracranial metastatic disease, drug deliver-system

## Abstract

Low-intensity MR-guided focused ultrasound in combination with intravenously injected microbubbles is a promising platform for drug delivery to the central nervous system past the blood-brain barrier. The blood-brain barrier is a key bottleneck for cancer therapeutics via limited inter- and intracellular transport. Further, drugs that cross the blood-brain barrier when delivered in a spatially nonspecific way, result in adverse effects on normal brain tissue, or at high concentrations, result in increasing risks to peripheral organs. As such, various anti-cancer drugs that have been developed or to be developed in the future would benefit from a noninvasive, temporary, and repeatable method of targeted opening of the blood-brain barrier to treat metastatic brain diseases. MR-guided focused ultrasound is a potential solution to these design requirements. The safety, feasibility and preliminary efficacy of MRgFUS aided delivery have been demonstrated in various animal models. In this review, we discuss this preclinical evidence, mechanisms of focused ultrasound mediated blood-brain barrier opening, and translational efforts to neuro-oncology patients.

## Introduction

Intracranial metastatic disease (IMD) is the most common type of brain tumors (1, 2) with over 20% of all oncology patients expected to have a metastatic brain lesion, and an annual incidence of 170,000 in the United States alone (1, 3–6). The rates of IMD are on the rise, which may be partially explained by improved imaging modalities facilitating earlier detection and prolonged survival of cancer patients due to advances in oncological care (7). Primary lung, breast and melanoma cancers are the most likely to metastasize to the brain, accounting for 67–80% of all brain metastases (3).

Surgery and radiation therapy are the cornerstones for management of IMD, with most intracranial metastases considered chemo-resistant (8, 9). The median survival period of untreated patients runs from the order of weeks to a few months, and can be prolonged to 4–6 months with the use of whole brain radiation therapy (WBRT) (10). For patients with a single brain metastasis < 3 cm in size, surgical resection or stereotactic radiosurgery (SRS) have shown survival benefit (11–14). The evidence and indications for surgical resection in patients with multiple brain metastases are much less established. Radiosurgery is favored for treatment of multiple lesions and, historically, patients with more than 4 lesions were treated with WBRT (15). However, SRS has become a viable option in this setting, as WBRT is associated with greater neurocognitive adverse effects (e.g., immediate memory, delayed memory, attention, and executive functions), and without significant added benefit in overall survival (16–21).

Chemotherapies that effectively treat the primary cancer and extracranial metastases remain largely ineffective for treatment of IMD (22). The function of the blood-brain barrier (BBB) and efflux transporters play a major role in suppressing the effectiveness of chemotherapies in the brain. The BBB excludes many chemotherapeutic agents from access to the brain, and the drugs that are able to penetrate may do so in insufficient concentrations. Another potential explanation for the ineffectiveness of chemotherapies in the brain is that IMD arises from chemoresistant clones (23). The primary cancer is often treated with chemotherapeutic agents, and thus only the chemoresistant clones metastasize to the brain. However, patients with IMD and are naive to chemotherapeutic agents continue to demonstrate decreased intracranial response rates compared to extracranial response rates, suggesting that chemoresistant clones alone do not explain this phenomena (23, 24). The BBB is, also, thought to confer an immune privileged microenvironment in the central nervous system, preventing access of surveilling immune cells to the tumor cells (25).

The BBB is a highly selective semi-permeable membrane formed by tight junctions between endothelial cells that primarily separates the circulating blood from the central nervous system (CNS). In addition to endothelial cells, the BBB is augmented by pericytes, astrocyte projections (also known as glia limitans), and neurons to provide biochemical support (26). The BBB is largely permeable to lipophilic compounds smaller than ~400 Da. The BBB is essential in protecting the CNS from circulating pathogens. At the same time, it is a key impediment for cancer therapeutics to effectively treat IMD. For example, doxorubicin, a common chemotherapeutic, is ~540 Da in size, albumin is 66.5 kDa in size, and most targeted or immunotherapies are of even larger size, such as trastuzumab at 148 kDa. These agents would have difficulty traversing a normal BBB. In addition, the penetration of therapeutics into the parenchyma is limited by the presence of p-glycoprotein 1 (P-gp) bound to the surface of endothelial cells. P-gp is responsible for efflux of chemotherapeutic agents, and is particularly abundant in cancerous tissue (27, 28). Thus, the BBB substantially limits the bioavailability of chemotherapies in treating IMD.

There is a pressing need for improved therapeutic delivery or effective circumvention of the BBB to improve the management of IMD with therapies that have been effective against the primary lesion. Existing methods to circumvent the BBB include convection enhanced therapy with intracranial injections or modification of the drug such as with nanoparticles to help penetrate the BBB. Convection enhanced delivery, however, requires implantation of intracranial catheter and results still in limited diffusion of drug from the catheter tip (29). Current nanoparticles and therapeutic modifications may also result in peripheral toxicity, such as unwanted accumulation in other end organs (30). The BBB permeability is also known to be increased by radiation therapy. In such a case it improves effectiveness of concurrent chemotherapy (31, 32). However, this approach is limited by the unpredictable temporal characteristics of radiation-induced BBB disruption and a radiation-induced injury to the surrounding normal brain tissue such as gliosis, necrosis, or demyelination (31). Furthermore, drugs that are delivered across the BBB in a spatially nonspecific manner can increase the risk to normal brain tissue.

Accordingly, a non-invasive, temporally, and spatially controlled BBB opening that is repeatable could significantly improve the management of IMD. Low-intensity MR-guided focused ultrasound (MRgFUS), in combination with intravenously injected microbubbles, fulfill these design requirements. In this review, we discuss the preclinical evidence of the circumvention of the BBB with low-intensity MRgFUS. Recent translational efforts and potential applications, along with critical areas for improvement.

## Focused ultrasound

Transcranial MRgFUS is an emerging image-guided, surgical modality that enables accurate steering of ultrasound energy into discrete targets within the brain. This technology utilizes a phased array of transducers to exert either thermal or mechanical effects on target tissue depending on the acoustic parameters, with higher intensity and frequency settings used for thermal effects. Currently, high-intensity MRgFUS operating at 650 kHz is approved by US Food and Drug Administration (FDA) for thalamotomy, an option for patients with essential tremor. At these parameters, ultrasound sonications rapidly result in temperature rise above 56°C, and well-circumscribed coagulative necrosis in the targeted region (33). In addition, sonications at the sub-lesional temperatures can result in transient neurological effects, to ensure accurate target selection.

Historically, Patrick et al. first found BBB disruption in the periphery of high-intensity focused ultrasound (FUS) lesions (34), with subsequent studies demonstrating BBB opening induced by low intensity protocols without damage to surrounding neuronal structures (35–37). Currently, clinical studies are conducted using a MRgFUS device operating at 220 kHz. MRgFUS opens the BBB primarily through two mechanisms: (1) disruption of the tight junction and (2) induced transcytosis. Cavitation, a biological effect of ultrasound, occurs through oscillation of gas bubbles formed within vessels after exposure to ultrasound energy, resulting into disruption of the tight junctions between endothelial cells, which has been shown via immunoelectron microscopy. This disruption is temporary and is restored after ~4 h (38). It permits the paracellular passage of molecules (38). There is also the evidence that the physical stress on the vessels leads to cellular changes that increase paracellular and transcellular transport of molecules across the BBB (38, 39), along with increased caveolins, an integral membrane proteins involved in receptor-independent endocytosis, and decreased P-gp visualized after FUS (38, 40, 41).

**Figure 1:**
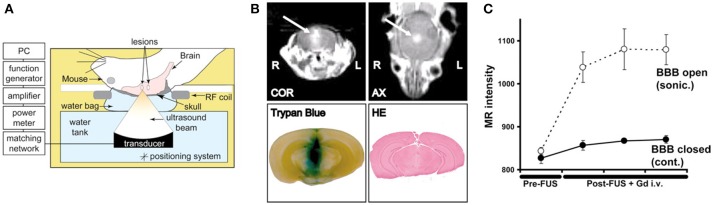
Demonstration of MR-guided focused ultrasound mediated blood-brain barrier opening in an animal model of intracranial breast metastasis. **(A)** Diagram of experimental set-up shows administration of ultrasound through the intact skull. **(B)** Representative example of focused ultrasound induced blood-brain barrier opening, as demonstrated by increased gadolinium (arrow) and tryphan blue extravasation. Hematoxylin and eosin stained tissue demonstrated preservation of gross tissue integrity and lack of macroscopic hemorrhage. COR, coronal scan; AX, axial scan; HE, hematoxylin and eosin. **(C)** The change in MR image intensity over time in the sonicated vs. non-sonicated regions. Bars represent standard deviation. Reprinted with permission from Kinoshita et al. PNAS 2006 (45), copyright 2006 National Academy of Sciences.

To further augment the cavitation process, exogenous microbubbles can be introduced into the blood system by intravenous administration ahead of sonication. The addition of exogenous microbubbles has been found to reduce the energy required to initiate cavitation by 100-fold, and increase the permeability lasting ~6–8 h. Sonications are typically initiated within half a minute of injection to allow sufficient circulation of the microbubbles (42). Consequently, ultrasound sonication can be made safer with a minimal injury to the surrounding tissue. The most commonly studied molecules are gadolinium-based contrast agents, which makes it easy to confirm the successful BBB opening using MR imaging due to the gadolinium now crossing the BBB and to assess the size of treated region (36, 43, 44) (Figure 1). Serial gadolinium scans showcased full closures of the BBB in 90% of cases at the 6-h mark post treatment, while the remaining rats displayed considerable decrease in the enhancement of the BBB opening and complete resolution at the 24-h mark post procedure (46).

Although overly high sonication powers can result in inertial cavitation and capillary damage, BBB opening is inducible at lower settings, at which results have been shown to be reproducible in large animal models (e.g., nonhuman primates) without any significant adverse events (47, 48). Successful weekly whole-hemisphere BBB openings for 4 weeks in elderly beagles was demonstrated using MRgFUS (49). This group, according to neuroimaging and histology analysis, reported no significant or enduring damage to any brain tissue targeted by MRgFUS. This discovery is significant evidence to support the safety of FUS administered treatments for brain metastases, as clinical utilization commonly requires several BBB openings for treatment. These MRgFUS parameters and study findings indicate its application-based significance that is capable of being translated to future clinical studies.

MRgFUS holds numerous benefits over other methods of drug delivery system. Paired with MRI guidance, FUS is capable of millimeter spatial accuracy of targeted regions within the brain, including the brain stem region (50). This precision allows targeted delivery of cytotoxic drugs only to abnormal tissue or specific areas in the tumor, which advanced imaging techniques can further help identify (51). Additionally, MRgFUS permits uniform delivery, in contrast to other intracranial treatments, such as convection-enhanced delivery. Lastly, the parameters of BBB opening can be adjusted by modulating ultrasound parameters to further customize treatment.

## Chemotherapies

Traditional chemotherapies for extracranial cancers have not generally been effective for brain tumors. A possible exception is temozolomide which is used for treatment of glioblastoma due to its limited side effect profile and central nervous system bioavailability. This is demonstrated by a group that used FUS-aided BBB disruption in a rat model to enhance temozolomide delivery to treat glioblastoma (52). In addition, another study displayed similar effects to improve drug delivery of temozolomide to treat a glioma in a mice model (53). Doxorubicin, an inhibitor of topoisomerase II, blocks DNA and RNA synthesis, and is effective in treatment of a broad range of tumors (54). Doxorubicin cannot cross the BBB to any appreciable extent and, as a result, demonstrates little effectiveness in treating CNS malignancies when administered systemically (55, 56). Further dose escalation is limited by cardiac toxicity. In preclinical studies, doxorubicin is effective against glial tumors *in vitro* and in animal models, and when administered intratumorally to patients via an Ommaya reservoir (57). While doxorubicin with FUS has predominantly been investigated in an animal model of malignant glioma, the results of significantly improved tissue concentration (e.g., 21 times in one study) and antineoplastic effects show promise for IMDs (58, 59). Further supportive survival data from the same group has recently been published and demonstrated a significant survival advantage in a rat glioblastoma model when using doxorubicin in combination with ultrasound-mediated BBB disruption. Delivery of doxorubicin to a brainstem was also recently shown to be feasible and safe for animals after histological and behavioral tests (50). Other chemotherapies investigated in conjunction with MRgFUS BBB opening in small (e.g., rat, rabbit) to large (e.g., nonhuman primates) animals include paclitaxel (60), methotrexate (61, 62), doxorubicin (50, 63, 64), cisplatin (65), bevacizumab (66), and carmustine (67) (Table 1).

**Table 1:** Representative change in therapeutic's concentration in tumor in sonicated relative to non-sonicated regions after systemic administration of therapeutic.

**Therapeutic**	**References**	**Approximate relative change**
Doxorubicin	Treat et al. (58)	21 x
Liposomal paclitaxel	Shen et al. (60)	2 x
Cisplatin-loaded BPN	Timbie et al. (65)	30 x
Liposomal methotrexate	Wang et al. (62)	9 x
Trastuzumab	Kinoshita et al. (45)	2 x
Interleukin-12	Chen et al. (68)	2 x
Bevacizumab	Liu et al. (66)	Range 5.7 x-56.7 x
Carmustine	Liu et al. (67)	2 x

*BPN, brain penetrating nanoparticles*.

## Targeted therapy

Technological advances and greater understanding of molecular biology have made an increased number of targeted therapies a standard of care for cancer patients. Targeted therapies address specific molecular aspects of cancer biology. Human epidermal growth factor 180 receptor 2 (HER2) is addressed when trastuzumab has been administered, and specific inhibition of mutated BRAF, a proto-oncogene is addressed when vemurafenib is administered. Trastuzumab has been found to be highly effective in controlling local and distal breast cancer lesions (45, 69, 70). BRAF inhibitors such as vemurafenib and dabrafenib are effective in extending the progression free survival of BRAF-mutant melanomas which are present in 50% of melanomas and is associated with significantly higher incidence of CNS involvement (71). The development of targeted therapies has been the cornerstone of precision medicine. Many targeted therapies have been approved for clinical use and may be used in combination to inhibit simultaneously multiple pathways which are important for tumor growth.

Trastuzumab is ~150 kDa in size, which makes it too large to pass through the BBB. In a rodent study, the tissue concentration of trastuzumab after systemic administration was undetectable (< 780 ng/g), whereas after sonication, the concentration increased to 3257 ng/g of tissue (45). This significant increase in trastuzumab concentration in tissue using FUS was further corroborated in a xenograft rodent model (70). Notably repeated dose of FUS greatly increased the concentration yield (70). In another HER2/neu-positive human breast cancer xenograft model, 6 weekly treatment of FUS plus trastuzumab led to what appeared to be complete resolution on MRI (69). The group where trastuzumab was administered along with FUS had significantly slower growth rate than controls. In another study of HER2-positive cells derived from cancer patients, 6 weekly treatments of trastuzumab and pertuzumab along with FUS led to a response in 4 out of 10 rats compared to none in the antibodies only group (72). These studies have paved the way for clinical translation in MRgFUS mediated BBB opening for patients with IMD.

## Immunotherapy

Immunotherapy directly helps or stimulates the patient's immune system to treat cancer. For instance, checkpoint inhibitors support T-cell surface receptor recognition and activation against cancer cells. Other types of immunotherapy include immunization, cytokines (e.g., interleukins), and cell therapy (e.g., CAR T-cell therapy) (68, 73, 74). Ipilimumab, a monoclonal antibody to T-lymphocyte-associated protein 4 (CTLA-4), another checkpoint in the immune system, is used in the treatment of unresectable and metastatic melanomas. These drugs have shown some preliminary efficacy in phase II studies in patients with IMD from lung cancer and melanoma, with ~20–30% 1-year survival rate. Several animal studies have established the safety and feasibility of delivering both cell-based (e.g., NK-92 cells) and cytokine (e.g., IL-12) in rodent models, with preliminary evidence of efficacy. Specifically, a repeated, biweekly treatment paradigm of NK-92 cells administered by MRgFUS BBB opening, resulted in long-term survival in 50% of animals injected with HER2 amplified tumors.

## Carriers

Once the delivered drug therapies have penetrated the BBB they are confronted by the extracellular space (ECS) of the brain, which extensively dictates and restricts the movement of the therapeutics in the brain. The ECS consist of mixed hydrophobic and electrostatically charged areas that comprises about 15–20% of the entire brain volume. Initially to penetrate the targeted brain parenchyma and deliver the appropriate drugs, brain-penetrating nanoparticles (BPNs) coated densely with poly(ethylene-co-glycol) (PEG) was explored, which has superior stability in the bloodstream (75). However, PEGylated BPNs results in reduced cell absorption or exchange through the BBB. Although it may potentially be used in combination with MRgFUS BBB opening.

A research group had recently explored this concept in rodents and reported the evidence of the successful first time use of MRgFUS and microbubbles with a biodegradable BPN platform which could penetrate and effectively transport therapeutic agents within the targeted areas of the CNS (76). It was also discovered that higher pressures of FUS modify the dispersal of the BPNs in the CNS, permitting more coverage and improving further the penetration within the targeted regions of the brain. Another study in rodent model of the breast IMD demonstrated substantial growth inhibition after one treatment of intravenously delivered PEGylated liposomal doxorubicin nanoparticles with FUS-induced hyperthermia without BBB opening (76). We may conclude that, the addition of carriers to existing therapeutic agents delivered through a BBB opening may provide some additional advantages for drug distribution in the parenchyma.

### Other applications of focused ultrasound

FUS creates a transient, targeted opening of the BBB that allows bidirectional communication between the systemic circulation and the central nervous system milieu. Shedding cancerous cells or cells' components is another possible result of the application of FUS. Blood-based analysis of circulating tumor cells, DNA, micro RNA, and extracellular vesicles hold the promise of improving upon histopathologic examinations or obviating the need for tissue biopsy (77). However, the challenges in these approaches lie in their uniform lack of sensitivity, with tumor DNA representing < 1% of total circulating DNA, and significant advance technology required for analysis (78). A proof of concept study in rat glioma model using fluorescent markers shows promise, but it remains to be demonstrated how this may be clinically applied (79). Finally, non-thermal ablation of tumor tissue using low-intensity focused ultrasound is being developed as a viable alternative to high-intensity focused ultrasound, where energy required to ablate large tissue volumes limits its safety and feasibility (80–82).

## Clinical applications and limitations of FUS in IMD

The feasibility and preliminary efficacy of focused ultrasound-assisted targeted delivery of cancer therapeutics have been demonstrated in various animal models. Clinically, there is now preliminary data regarding safety and feasibility of focused ultrasound BBB opening with co-administration of carboplatin in patients with gliomas (83). Doxorubicin and temozolomide delivery studies using MRgFUS are underway for neuro-oncology patients (NCT02343991, NCT03322813). For other neurological disorders, a pilot study of MRgFUS BBB opening in patients with Alzheimer's disease (84) was recently reported to have demonstrated safety and feasibility. Finally, a study for patients with amyotrophic lateral sclerosis is also underway (NCT03321487).

Notwithstanding the potential advantages to MRgFUS for therapeutic delivery for patients with IMD, there are important limitations. They include the need for pre-procedural removal of hair, the substantial operating time of the procedure, and the use of a stereotactic frame, which may represent limitations for widespread utilization and tolerability. In addition, clinical experience with MRgFUS induced BBB opening is preliminary with side effect profile (e.g., microhemorrhage, ischemia) in human subjects still to be characterized. Furthermore, essential technical data is urgently needed regarding the feasibility in tissues of various interstitial pressures, tissue, and vascular properties, and pathologies, such as in the case of peritumor edema. Future modifications of this technique may include controller based on acoustic feedback will likely significantly shorten operating time while preserving the uniformity of BBB opening (85). Finally, the specific treatment protocol and dosing remain to be elucidated for each anti-neoplastic agent. MRgFUS will most likely be most beneficial for patients with IMD and relatively well controlled systemic disease burden. MRgFUS is a drug delivery platform, where in the age of precision medicine and with the increasing availability of advanced imaging, it opens up exciting opportunities for induction of the precisely targeted delivery of drugs to the brain. Although still in the early investigational stages, this minimally invasive technology for targeted BBB opening has the potential to revolutionize the care of neuro-oncology patients.

## Author contributions

All authors listed have made a substantial, direct and intellectual contribution to the work, and approved it for publication.

### Conflict of interest statement

NL has served as paid consultants on expert steering committees for the Focused Ultrasound Foundation. The remaining authors declare that the research was conducted in the absence of any commercial or financial relationships that could be construed as a potential conflict of interest.
